# Obstetrics and Obstetrical Surgery

**Published:** 1899-09

**Authors:** Eric Vondergoltz

**Affiliations:** New York


### HOMŒOPATHIC PHYSICIAN,

A MONTHLY JOURNAL OF

HOMŒOPATHIC MATERIA MEDICA AND CLINICAL MEDICINE.

"If our school ever gives up the strict inductive method of Hahnemann, we are lost, and deserve only to be mentioned as a caricature in the history of medicine."—CONSTANTINE HERING.

Vol. XIX. SEPTEMBER, 1899. No. 9.

### OBSTETRICS AND OBSTETRICAL SURGERY.

By Eric Vondergoltz, M. D., New York.

(Synopsis of 565 Obstetrical Cases.)

Recent publications show, on one side, that the expected benefit from antisepsis is not visible in private practice as compared to the results in hospitals regarding morbility and mortality of the mothers.

On the other side, many authors claim superior results from a strict asepsis rather than from antisepsis.

Therefore, I examined my records carefully to find why these opposed, or seemingly opposed, opinions could be sustained by a considerable literature.

The rational manipulation of a hospital resembles the regularity of a well-adjusted machine, where each part has its own function. This, generally, will not be witnessed in the private home during confinement.

There prevails a certain anxiety and consequent disorder, and almost everything depends on the physician, that he must take entire management and control in his own hands.

If in each case the physician will follow a well-defined way of his own, an established routine will give a good result, even in encountering the most daring obstetrical operations.

In looking over the various works on gynæcology and obstetrics, we will find on every page of a gynæcological book the detail with which the single and diminutive steps of an operation are described—even when referring to the smallest operation of minor surgery, while we find in obstetrical books merely a wholesale dealing with the antiseptic and aseptic preparation of an obstetrical operation. Perhaps the writer is too anxious to explain most thoroughly an operation that more or less will be asked for of every physician. At the beginning of my practice I strictly followed the rules of the management of Basle; T. T. Bischoff, late professor of obstetrics and gynecology, wrote, in 1875, an article concerning his antisepsis and of his results. Although his paper is now twenty-four years old—and, generally, a medical thesis loses in the lapse of time—the *punctum resistentiæ* has remained the same. I will try to translate his words:

"Try to overcome and remove, as much as possible, all probable cause of any endangering decomposition from the vaginal tract before any lesions by the delivery could happen."

The results obtained by Dr. Bischoff in 1875, when the technique of antiseptic surgery was by no means such a safe one as to-day, have been so favorable that the publication of his article, "Prophylaxis des Puerperalfiebers," was the result.

But I soon found the importance of varying these rules on account of the many differences in the habits of the people, so that I could more easily meet every arising emergency.

If called for and arrived at the home of the parturiens, I see, first, if all my previous orders have been followed, that the water is boiling in clean vessels, that towels, bed-clothes, and bed are prepared according to my wishes, that the patient has donned such garments as I advised during the engagement call.

Then my instruments (forceps, needles and needleholder, sutures, scissors and ligatures) are immersed in a boiling two per cent. Lysol solution—if needed or not. The hands and arms cleaned to the elbows, as also the genitals of the parturiens preceding the examination. Then I give a very scrupulous washing and vaginal irrigation with one-half per cent. Lysol solution, before and after the examination. If the right time for the delivery is not yet at hand, everything remains in order. I go home, and call again as labor progresses. If my first examination does not reveal the necessity, no further examination will be made, and, therefore, no further vaginal irrigation is necessary, otherwise every examination will be followed by a cleansing as vigorous as the first. It must be understood that the evacuation of the bowels and bladder has been previously demanded, so that no interrupting accident is to be expected.

The authorities of a management of labor without vaginal examination and without prophylactic vaginal douches, like Winkel, Gusserow, Mermann, Tippins, and Köld are clinical obstetricians, and so the management can, perhaps, be made safe by an enforced asepsis, which last, but not least, will prove a derivation of antiseptic cauteles—but only effective in their own institutions.

Not every one is such an obstetrician that he can neglect the vaginal examination.

Besides this, we have to consider the surroundings in the private home. There are few stained floors—we are endangered in our asepsis by carpets, rugs, papered walls, and pictures.

All this, certainly, will not confirm the abstinence from antiseptic precautions, especially as we have to deal so often with dark bedrooms, where the germ-destroying influence of air and light cannot penetrate.

The value of the vaginal injection has to be regarded from the side of the technique. Here we have to consider two points—the instrument and the manipulation.

The first demand, in my eyes, is that every physician shall have his own syringe, as also his own forceps. The syringes of the household cannot be used, as they are not clean for the use of the surgeon. In referring to the manipulation, a simple syringing is ineffective; this syringing has to be done as an effective cleaning; and this is a scrubbing and washing, but, of course, with absolutely and unquestionably clean and aseptic, *vice versa* antiseptic, fingers.

All that material of the advocates of asepsis lacks one point—the manipulation has not been explained; and then I do not find the necessary absolute confirmation of no puerperal infection. One especially, Mermann, had under this treatment "no case of severe puerperal infection." . . . "One woman only was discharged on the eighteenth day."

As soon as the delivery draws to the end, I bring the parturiens on the transverse bed on a perineal pad; the body is protected by a blanket and the feet are dressed in clean stockings.

Two relatives or friends are supporting the legs, so that the woman does not feel uncomfortable. I take my place in front of the patient. If necessary, the external genitals will be again cleaned and the delivery goes on.

One of the bystanders is instructed to receive and hold the new-born till the ligaturing and cutting of the cord is done.

In this position the patient will be kept till she is dressed and put back in her clean and unsoiled bed. All water, blood, and placenta will be received in a pail, slipping down the pad. All this does not take so much time, as where everything is spoiled, and the woman remains in her water and blood, perhaps, for a very long time. The after-treatment consists of a vaginal douche of two-tenths per cent. Lysol solution, twice a day, up to the fifth day. Every three hours the Lysol gauze, which protects the vulva, will be changed by the attendant, who, before touching the woman, has to clean her hands with a one-half per cent. Lysol solution.

One of the most important questions is the preparation of the physician; and this preparation will be a success only if the instruments and the whole outfit are subjected to the most vigorous application of antiseptic rules.

The obstetrical bag must be used only for confinements. The instruments must be cleaned after each use and packed away fully sterilized. My outfit consists of forceps, of medium size; needles and needleholder; pair of scissors; two traculum-forceps, and Edebohl's speculum and legholders; curettes, and an intrauterine catheter; also, silkworm and cord ligatures and fifteen yards of prepared two per cent. Lysol gauze; three pairs of long (tuit) and six pairs of short (déan) hœmostatic forceps, and two straight and one curved blunt-pointed knives. These legholders are the best help for restoration of the cervix and perineum, where the assistance of the relatives may prove very unsatisfactory.

My failures in the first years of my practice, especially in the restoration of cervical and complete perineal tears, were readily overcome when I made these operations on a table. This can be done quickly if everything (as I quoted in a former place) is kept ready for eventual use.

I must add that for emergency cases I fill another bag with the required instruments. These instruments, so far, have proved absolutely reliable as being always in a state of perfect cleanliness (after each use they are boiled in soda-suds, polished in Lysol solution and dried by heat). Then an immersion in ten per cent. Lysol solution shortly before the operation will be sufficient. The high grade of Lysol and the slipperiness will be removed by immersing in plain boiled water when taking for use.

Such a handling of a confinement will seem, at the first glance, polypragmatic, but I believe that such a polypragmacy will benefit also the physician, who has to encounter an instrumental delivery with following operations. So the confinement will be turned easily into a safe channel, well prepared concerning patient, room and attendance, with a good result.

If such vigorous antiseptic cleanliness has been achieved, then we will have better results in cases where the most important operations, as accidental, during confinement have to be performed.

Finally, I have to add that my patients receive instructions for immediate preparation of the body, nourishment, etc., the same as in cases of operations. I am convinced more and more that preparations in this line regarding diet, evacuation of the bowels, learning to pass the urine in a reclining position, spare many inconveniences.

The nurse is the only factor that could increase the cost. In every household we must have a woman to do the housework during the confinement, so the expense will not necessarily be increased.

The seeming sacrifice rests with the physician; but only at first. After practising such self-imposed rules every one will find a saving of time, where, generally, we hear that these confinements disturb the regular day's routine.

I believe it to be my duty to explain why I do not follow the general rule, to carry with me those obstetrical instruments advised by our great teachers. To take these complicated and so very seldom needed instruments will not improve the absolute cleanliness demanded, and, again, the examination of the pelvis during the engagement call will suggest the eventual use.

*The engagement call and examination is the basis of successful antiseptic midwifery.* Then, when called in by urgency at the last moment, the antiseptic or aseptic management is utopian.

I am convinced that nobody will undertake the performance of any operation, or treatment, neither give a prescription, without an exact examination leading to such an advice. It sometimes occurs that a lady asks a medical man through her husband to attend her confinement—perhaps, only to save her the examination—the feelings of the patient being too sensitive. Dr. Lusk speaks, in his book, *Science and Art of Midwifery*, about this false modesty as mock modesty in reference to the conduct of normal labor, when rupture of the water-bag is imminent; I would like to draw the line broader and call the whole modesty mockery, as soon the earnest work of the accoucheur is hampered, either in preparation or in conducting the labor. We must not forget that this modesty is a result of the present existing fashion. To bow to such a fiction under certain conditions will pay poorly. All mock modesty can be overcome by honest work on the part of the accoucheur.

If aggressive antisepsis will be carried out a following occurrence will belong to the past.

A midwife has in charge a slowly progressing confinement for twenty-eight hours, finally a doctor is called in, on account of atony of the uterus and to save the infant. The woman is easily delivered by the forceps. The doctor has his highly polished and plated instrument boiled and carbolized, hands clean and disinfected.

Two and a half hours later the patient gets chills, a high temperature of 105 degrees sets in, distension of the abdomen, vomiting, facies Hippocratica, etc. Who is the culprit? Of course, the family charges the doctor. This juncture of the darkest and most regretful uncleanliness can be witnessed very often. I know that many physicians are very cautious in accepting calls for aid in such cases.

In addition, I may be allowed to make some propositions to frustrate the evils lurking around the lying-in bed:

1. That during the engagement call the examination of the pelvis will be conducted with the utmost accuracy for judging the obstetrical aid so far as possible.

2. To instruct the engaging party as to cleanliness concerning person and surroundings, under the impression that so mother and child will be best provided.

3. That the medical profession shall try to enlarge and educate their clientele to absolute cleanliness as the preparatory step to antisepsis and asepsis.

4. That the medical profession shall try all means to bring antisepsis and asepsis to a better understanding of the midwives, for greater safety in regard to obstetrical operations.

5. That the preparation of the physician will be carried out in a strict and pedantic way—always regarding the individualism of the concrete case.

The plea of this whole essay in regard to the obstetrical work of the practitioner, for a minute system prominently in antisepsis, is caused by the following observation, that as everybody must learn and broaden his experience by the different observations, many trials of the beginner in the direction of a certain operation will be less destructive if finally given up as impracticable. It is easy to teach that an operation shall never be undertaken if all pro and contra are not fully understood; this happening will be on the one side easier avoidable, and on the other side less dangerous.

Easier avoidable, as such a preparation gives chance to observe every particular moment, and, second, that this trained management gives better chances for execution of operations, otherwise well chosen, but impossible, as the patient does not occupy the favorable position, etc.; as for instance, a friend of mine called me to help in a forceps delivery. The physician was exhausted. The patient was, on my arrival, under the influence of Chloroform on the transverse bed, the legs were kept in position on chairs by the attendants. A hasty examination showed forceps in the right position—only the patient was lying with the buttocks too far from the edge of the bed, so that there was no place to work the forceps! As the patient was brought well down, and the legs were well bent and adducted to the abdominal parietes, the forceps delivery was the easiest imaginable, to the surprise of my friend.

All this must be considered more or less important, but at times such management in all its details will show the profits.

If, on the other side, any operation must be given up on account of being ill chosen, while everything was done in a perfect antiseptic way by means of far-going preparations, such an abandoned manipulation has no bearing; different trials of version will be harmless, where finally an embryotomy will confine the mother.

As a former Allopath, I was anxiously looking for the benefits of Homœopathy at the bedside. My observations on this subject are abstracted from experiments. On the one side, I felt myself perfectly confident as to the surgically conducted confinement; on the other side, I felt a need for a substitute for my surgical activity, remembering fully that nature had not created a physician or surgeon standing at the bedside of the first parturient woman on earth.

Besides the known remedies in their excellency regarding pains, expulsion, etc., I will enumerate some which were tried by me (only on the supposition of correspondeney in their activity in other regional complaints of the body).

*Apis-mel.*, excellent in reducing the swelling after primary trachelorrhaphy and perineorrhaphy with too tight sutures, where either a gangrene was to be expected or where the sutures had to be removed.

*Arnica-mont.*, very sensitive and hysterical primiparæ; to overcome quickly the nervousness, etc. The patient regains more easily her equilibrium, and everything begins to go on as if nothing had happened.

*Belladonna*. I prefer to give Bel. if to the third day after delivery the reinvolution of the uterus is not well established. We always can find more Belladonna symptoms—as I personally have found—but this landmark, found by the daily palpation of the abdomen, is sufficient to prevent in time any perimetritis and parametritis, going out from this slow reaction of the uterus.

*Hypericum-perfol.* If the whole system is affected, like by an electric shock.

In regard to the question: Where comes Homœopathy during labor, and what position to take regarding instrumental delivery? I am now following this maxim: I wait and follow up with homœopathic remedies as long as I do not see exhaustion of either the parturiens or of the child (heart-sounds, etc.).

The pride of the obstetrician shall be not the final expulsion of the fetus, dead or alive, but to terminate a confinement by judicious application of reliable means.

Such an act of a homœopath as the following citation is unworthy. It seems like the man who lets a child fall into deep water, waiting till it is nearly drowned, then takes it from the water and *recalls it to life by proper care.*

Richard Hughes, L. B. C. P., Edin., writes, in his *Manual of Therapeutics,* verbatim:

"In the case of a woman, twenty-six years of age, in her first labor, in whom the sacro-pubic diameter of the superior strait did not offer more than two inches and a half, I had the patience to wait for seventy-two hours the natural efforts of labor. The head being in the first position, at the end of the second day it began to engage in the superior strait. At the end of the third day the pains slackened very much; the woman became very feeble, was pale, exhausted, and had lost all hope. I put Secale-cor. 30 into a glass of water, and gave her a teaspoonful at 11 o'clock in the evening. Some minutes after she fell asleep and slept very quietly for three-quarters of an hour, when, awakened by a violent pain, she made a courageous effort, and two hours after gave birth to a child, pale, in a state of asphyxia, but which was recalled to life by proper care."

Every rational obstetrician will (as I firmly believe) approve the following statement of mine, and so give a damaging critique of such a doing:

If the stadium of expulsion has begun and the child is not born between two to four hours after, and the remedies, etc., could not effect the expulsion, the physician shall extract the child by means of the forceps, etc.

I ask, Are these waiting physicians (seventy-two hours!) so absolutely sure to be able to cope with a sudden rupture of the uterus? Do not these physicians think that a gangrene by pressure will be easily effected by their "patience"?

I am a convinced homœopath, and have seen many deliveries effected by my remedies, where, as consulted physician, I was preparing my instruments for any occasion. It is my custom to give, after a few questions, one dose of the indicated or needed medicine, to see, perhaps, the effect, during that time necessary for my preparations. If then I am convinced that the remedy will effect the expulsion, I am homœopath enough to wait; and I am glad to state that my trust in Homœopathy did not fool me.

On the other side, if called for help to a bed of a parturiens in pains for longer than from six to ten hours, I hastily give the necessary medicine, arrange my preparations, and if now ready for action, and the remedy without result, I put it down as an absolute duty to finish the confinement as speedily as possible under Chloroform!

I am decidedly against the absolute medical treatment, where one of the easiest and simplest manipulations will bring a quick finish.

The placenta and velamina if not expelled in from thirty to sixty minutes must be taken by the obstetrician.

The residua of abortions must be removed. To wait, to give medicines for weeks—only not to interfere—is not what Hahnemann understood by Homœopthy.

The post-partum hemorrhage and the severity of the after-pains of a multipara—occuping such a great space in homœopathic threapeutics is, after my observation, the result of too much medical and too little judiciously mechanical help—in a mostly mechanical field.

Except in hæmophiles, I hardly know the danger of uterine hemorrhage; hemorrhage is always, in my opinion, proof of retention of some part of the placenta or a deep laceration of the cervvix, etc. Curette and a few sutures will be the real remedy.

The too-severe after-pains in multiparæ is a proof of some faults in the management, as we will mostly observe these painful after-pains in multiparæ, where a slow reinvolution takes place; this is only the reaction of something wrong during the management of one of the second or third periods.

We never must forget that the manifestation of the minimal sepsis will sometimes not be recognized as sepsis and result of sepsis, but something different.

This is the result of my observations during the management of five hundred and sixty-five obstetrical cases, as compared with the management of other obstetricians without such absolutely decided antisepsis. It may seem overdone, such a management, but the following case teaches me to remain firm in my doings.

Mrs. T. G., æt. 34. eighth part.—I was called, July 11th, 1895, at six o'clock P. M. The fetal head well down, cervix began to thin. At eight o'clock A. M., July 12th, the water broke. The pains were till then very good, so that after this last event I congratulated the husband (a brother-physician) that the much dreaded confinement would turn out a self-development. But this prognosis was erroneous, as suddenly the cervix began to get rigid—and nothing would help. Finally, at twelve M., I sent for my assistant to give the anæsthetic. Meanwhile the patient began to get delirious. Chloroform was administered, and the patient, on the transverse bed in the exaggerated lithotomy-position, was scrupulously cleaned and washed; the very narrow and œdematous cervix was incised right and left, the blades of the forceps introduced and the head extracted with all care not to make any larger laceration. The narcosis was so splendidly given that I was not hindered in anything. The cord was wound twice around the neck of the child and so too short, thus giving the real cause of this delaying labor. The cord was cut immediately, the placenta already in the vulva, but the velamina were retained. I went in the uterine cavity with my hand and brought it down in one piece. What I am always doing as precaution I did in this case again. I curetted with a large, dull placentar curette, and rinsed the cavity with hot one-half per cent. Lysol solution. A small superficial perineal tear was then closed. Everybody witnessing the labor was astonished to see the recovery.

More than this, the patient made a quicker and an absolutely uneventful puerprium, no fever, no distention of the abdomen, no severe after-pains, no protracted lochiæ, which had been features of all her former regular and unaided self-developments.

The baby was in danger, the mother was exhausted. Was I there to wait? Was not my action proved by the result?

The reason I cite this case is the circumstance of the difference in the subjective and objective signs of her former and of this last confinement.

### LITERATURE.

Doederlein, Archiv für Gynaecologie, Vol. XXXIV, p. 111.

Frommel, Deutsche Med. Wochenschr., 1892, No. X.

Hofmeier, Deutsche Med. Wochenschr., 1891, No. XLIX.

Mermann, Centralblatt für Gyn., 1892, No. XC.

Leopold & Goldberg, Deutsche Med Wochenschr., 1892, No. XIII.

Steffeck, Zeitschrift für Gebartshilfe, Vol. XV, p. 395.

H. T. Garrigues, Am. Journal of Med. Science, 1889, Vol. XVIII, pp. 109–128

Pippingskjöld, Centralblatt für Gynaeckologie, 1891, p. 680.

### SECOND PART.

The foregoing pages contain the abstract of the following histories, which shall show the applied operations, technical points and final results; on the other side shall be shown the difficulties which the obstetrician encounters regarding antiseptic cleanliness, handiness and readiness of assistants, etc.—absolutely indispensable for good surgical work.

The material of these five hundred and sixty-five cases is distributed in the following manner:

| | OWN PRACTICE | CONSULTATION |
|---|---|---|
| Self-development, | 225 | 225 |
| Instrumental Operations, | 73 | 107 |
| Manual Operations, | 19 | 76 |
| Abortus, | 17 | 48 |

MORBILITY AND MORTALITY.

| | OWN PRACTICE | CONSULTATION |
|---|---|---|
| Morbility of mother, | . . | 13 |
| Mortality of mother, | 3 | 4 |
| Morbility of children, | 7 | 15 |
| Mortality of children, | 3 | 19 |
| Still-births, | 4 | 21 |
| Abortus, | 17 | 48 |

INSTRUMENTAL OPERATIONS.

| MODUS OPERANDI | OWN PRACTICE | CONSULTATION |
|---|---|---|
| Forceps on the head, | 42 | 68 |
| Forceps on the breech, | 25 | 10 |
| Traction-forceps (head), | 3 | 17 |
| Cephalotribe, | 1 | 2 |
| Embruley, | . . | 4 |
| Symphysiotomy. | 2 | 6 |
| Forcible dilation of the cervix, | 41 | 11 |
| Episiotomy, | 27 | 19 |
| Tamponade of the uterus, | 15 | 9 |
| Curettage for abortus, | 17 | 48 |
| Curettage for retentio placentæ, | 22 | 72 |
| Trachelorrhaphy, | 51 | 32 |
| Perineorrhaphy, | 75 | 105 |
| Tears in the vaginal laquears, | 22 | 30 |

MANUAL OPERATIONS.

| MODUS OPERANDI | OWN PRACTICE | CONSULTATION |
|---|---|---|
| Version and Extraction of fetus, | 19 | 76 |
| Manual Detachment and Extraction of placenta, | 49 | 52 |

[TO BE CONTINUED.]

